# Microcontroller-Based PUF for Identity Authentication and Tamper Resistance of Blockchain-Compliant IoT Devices

**DOI:** 10.3390/s23156769

**Published:** 2023-07-28

**Authors:** Davor Vinko, Kruno Miličević, Ivica Lukić, Mirko Köhler

**Affiliations:** Faculty of Electrical Engineering, Computer Science and Information Technology Osijek, Josip Juraj Strossmayer University of Osijek, Kneza Trpimira 2B, 31000 Osijek, Croatia

**Keywords:** authentication, blockchain, internet of things, physical unclonable function

## Abstract

Blockchain-based applications necessitate the authentication of connected devices if they are employed as blockchain oracles. Alongside identity authentication, it is crucial to ensure resistance against tampering, including safeguarding against unauthorized alterations and protection against device counterfeiting or cloning. However, attaining these functionalities becomes more challenging when dealing with resource-constrained devices like low-cost IoT devices. The resources of IoT devices depend on the capabilities of the microcontroller they are built around. Low-cost devices utilize microcontrollers with limited computational power, small memory capacity, and lack advanced features such as a dedicated secure cryptographic chip. This paper proposes a method employing a Physical Unclonable Function (PUF) to authenticate identity and tamper resistance in IoT devices. The suggested PUF relies on a microcontroller’s internal pull-up resistor values and, in conjunction with the microcontroller’s built-in analog comparator, can also be utilized for device self-checking. A main contribution of this paper is the proposed PUF method which calculates the PUF value as the average value of many single PUF measurements, resulting in a significant increase in accuracy. The proposed PUF has been implemented successfully in a low-cost microcontroller device. Test results demonstrate that the device, specifically the microcontroller chip, can be identified with high accuracy (99.98%), and the proposed PUF method exhibits resistance against probing attempts.

## 1. Introduction

There is an ongoing trend of continuously increasing the number of IoT devices and the data they generate. As the number of IoT devices grows, various use cases emerge that rely on the data from the IoT ecosystem. One crucial requirement is the ability to trust IoT devices and their data. These use cases span different industries, including the Internet of Vehicles [1], automotive systems [2], smart agriculture [3], food-chain traceability [4], and IoT security in general [5].

Blockchain technology is primarily utilized as a tamper-proof platform for IoT data [6,7], especially in wireless sensor networks [8], to establish data trust [9]. Numerous papers have reported on the use of blockchain as a security feature for IoT [10,11,12,13], even in sensitive use cases such as medical [14] and industrial [15], where confidentiality, integrity, and data availability must be ensured [16].

While blockchain technologies can provide a safety net for IoT data through stored proofs, they cannot guarantee the integrity of the data at its source. In other words, they cannot ensure the trustworthiness of IoT devices or verify their authenticity. Blockchain can compare previous and current records of an IoT device, but it cannot guarantee that the device is not counterfeited or cloned.

Some solutions attempt to address this issue from the blockchain side by proposing novel consensus algorithms for IoT applications [17] and blockchain-based IoT authentication [18]. More comprehensive solutions involve additional stakeholders and aim at metrology applications [19,20].

All blockchain-based solutions for tamper-proofing and authentication assume that tampering and false authentication are rare events, detectable through anomalous behavior compared to previous records stored on the blockchain. However, IoT devices’ deployment scenarios, operating principles, and communication methods make them vulnerable to attacks, tampering, and manipulation [21].

One way to ensure that IoT devices cannot be counterfeited is to use devices that support secure elements [22], such as ARM TrustZone [23]. Secure elements facilitate the authentication of IoT devices and establish data trust between the devices and the blockchain [24].

Various methods are proposed for tamper-proofing devices. Some focus on tamper detection, such as acoustic [25] and optic [26] based solutions. Other approaches propose tamper-resistant methods, for example, by employing highly volatile encryption keys [27] that get destroyed if the device is tampered with, rendering the device data unrecoverable. Probing detection methods are also discussed to protect against tampering and invasive attacks [28,29]. One common solution against tampering is the use of tamper-proof enclosures, also known as envelopes or shields, which are designed to monitor their integrity [30], temperature changes [31], proximity of foreign objects [32], and casing openings [33].

### 1.1. Motivation

Solutions for identity authentication and tamper-proofing of IoT devices that rely on additional secure elements and tamper-resistant enclosures tend to be expensive, which is unsuitable for low-cost and low-power devices and sensors that constitute a significant portion of the IoT ecosystem. For such devices and sensors, a cost-effective solution is required to prevent illegal copying and the leakage of important data through theft, stealing, and hacking [34,35].

One such affordable solution is the use of PUF technology. PUF technology leverages the unique physical properties of each device, which can be observed as a fingerprint of the IoT device. PUF-based solutions [36] promise to provide lightweight physical identities for authentication in IoT devices [37]. By employing PUF technology, the security level of IoT platforms can be significantly enhanced [38], particularly for resource-constrained IoT devices [39,40] in use cases where device authentication is essential, such as blockchain-based IoT applications [41].

As an emerging physical security mechanism, PUFs can reduce the overhead of cryptographic computations and effectively resist physical attacks [42] and device cloning [43] by binding the data to the device fingerprint [44]. In a blockchain environment, while the blockchain ensures the legitimacy of the IoT device and data, PUF technology safeguards the IoT device against counterfeiting and tampering [45].

In recent years, several prominent use cases have emerged that combine blockchain and PUF technology synergistically. These use cases encompass general security and authenticity aspects, such as secure IoT applications [46], multi-server authentication protocols [47], secure firmware updates [48], mutual authentication schemes [49], and smart home applications [50]. More specific use cases relate to the Internet of Medical Things [51] and Wireless Medical Sensor Networks [52], the Internet of Vehicles [53], vehicle-related technologies [54,55], supply chain provenance [56], and product tracking [57].

Different types of PUFs have been proposed and evaluated. The fingerprint of each IoT device can be generated based on capacitance or resistance values due to a coating layer of the device, device-specific drain voltage values of CMOS transistors, random differences between two delay paths, or random bits from an unstable SRAM state [58]. These physical values vary from device to device due to manufacturing variability and are used to derive a unique identifying key for each IoT device. More complex PUF examples include ring oscillators [59], XOR logic gate meshes [60], and flip-flop logic circuits [61].

PUFs based on the manufacturing variability of integrated circuits (such as microcontrollers, SRAM memory, etc.) can be employed to verify the identity of the integrated circuit (IC). To extend PUF to the PCB level of the IoT device, a resistor-capacitor (RC) PUF [62] can be used in conjunction with IC-level authentication to provide a board-level fingerprint [63].

There are two main authentication methods using PUF technology. The first method is based on the PUF value, which can be used as a cryptographic key, or its hash can be sent to the blockchain for verification [64]. The second method is based on the PUF function, where a challenge is given to the PUF function (e.g., a sequence of signal pulses), and the PUF function returns a response (e.g., a sequence of pulses, voltage waveform, etc.) [65]. The second method requires a third party that stores valid challenge-response pairs [66]. While challenge-response PUFs are considered strong PUFs, they are vulnerable to Machine Learning attacks [67], which can model PUF behavior based on collected challenge-response pairs [68].

Considering this, device verification based on PUFs should be performed locally on the device itself to avoid third-party storage and potential device ID counterfeiting using Machine Learning techniques. An approach where the device performs an integrity self-check is presented in [69] and can be expanded to mutual authentication [70].

### 1.2. Main Contributions

This paper focuses on an RC PUF that combines the fingerprint of both the PCB and the IC. The main contribution of this work is the development of a PUF that can be utilized for device self-checking. In other words, the PUF decodes the program code during device power-up or periodic device authentication. To enable this functionality, the firmware must be securely deployed on the IoT device. Subsequently, the PUF is used for authentication each time the device is utilized.

The proposed PUF offers a low-cost hardware solution that imposes minimal overhead on microcontroller resources and PCB design. PUF values rely on measurements of analog quantities, which are susceptible to measurement noise. Consequently, there may be limited distinction in PUF values between devices. To address this issue, the proposed PUF employs averaged batch measurements, significantly enhancing the uniqueness and precision of the PUF by two orders of magnitude.

Section 2 of the paper comprehensively describes the proposed PUF, offering insights into its design, architecture, and underlying principles. This section aims to provide readers with a thorough understanding of the PUF’s operational mechanisms and distinguishing features.

In Section 3, the measurement results obtained from practical experiments are presented and thoroughly analyzed. This section highlights the performance of the proposed PUF in real-world scenarios, discussing factors such as accuracy, reliability, uniqueness, and any observed limitations. The analysis provides valuable insights into the PUF’s behavior and performance characteristics, enabling researchers and practitioners to assess its practical feasibility and effectiveness. This section may include potential applications, areas for further research, and comparisons with existing PUF approaches.

The paper concludes with a Discussion section, where the implications of the research findings are discussed in a broader context. It serves as a platform for drawing conclusions based on the presented results and encourages further discourse on the proposed PUF and its implications.

Lastly, the paper includes a list of References, providing proper citations for the sources and works referenced throughout the paper. These references allow readers to delve deeper into related research and gain a broader perspective on the field of study.

## 2. Proposed PUF for Identity Authentication and Tamper Resistance

Strictly speaking, a PUF should take inputs and generate corresponding outputs. However, nowadays, the term “PUF” is often used as a general term for any method that provides hardware-based security relying on the physical properties of a device. This paper describes a PUF method that leverages inherent manufacturing variations in microcontrollers. By measuring and analyzing these physical properties, a PUF can generate a unique identifier or response that serves as a hardware-based fingerprint for the device. Therefore, the term “PUF” refers to the device fingerprint in this paper.

Each low-cost IoT device is built around a microcontroller. This section explains how to achieve an IoT device’s identity authentication and tamper resistance using a PUF. The proposed PUF is a chip-level and board-level PUF, which identifies a specific microcontroller on a specific PCB. It can detect if the microcontroller is replaced, and it can also detect if the “correct” microcontroller is placed on a different PCB, i.e., a different IoT device.

**Figure 1:**
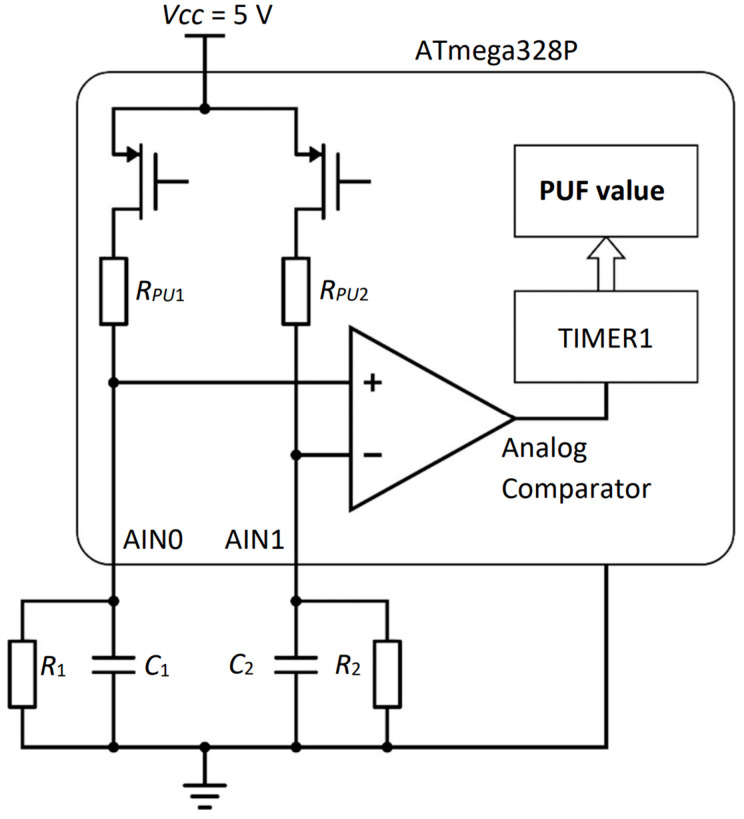
Schematic of proposed PUF.

The schematic of the proposed PUF is shown in Figure 1. The PUF is based on the internal pull-up resistors of the microcontroller (*R_PU_*_1_ and *R_PU_*_2_) and an *RC* network (*R*_1_*C*_1_ and *R*_2_*C*_2_).

Identity authentication is based on the PUF value, which is determined by the resistors’ physical (analog) values, capacitors, power supply, and current consumption of the microcontroller’s analog comparator. The proposed PUF is designed for low-power, low-cost microcontrollers that lack additional security features, such as a secure chip or cryptographic co-processor. To demonstrate that the proposed PUF can be used for identity authentication and tamper resistance even on resource-constrained devices, it was implemented and tested on an Atmega328P microcontroller.

All microcontrollers have digital I/O pins, and for each I/O pin, a pull-up resistor can be enabled when the pin is used as an input pin. The pull-up resistor value is not critical and has a large tolerance. For the Atmega328P microcontroller, the pull-up resistor values (*R_PU_*_1_ and *R_PU_*_2_) range from 20 kΩ to 50 kΩ. Together with two external RC networks (*R*_1_*C*_1_ and *R*_2_*C*_2_), it forms an analog circuit whose response strongly depends on the selected components.

In the Introduction, various PUF methods and approaches were discussed. Our proposed PUF combines two methods: imperfections in the silicon die during chip manufacturing (values of internal pull-up resistors) and the time response of an *RC* network. *RC* networks are utilized in two different ways. First, the device measures the PUF value and uses it locally for self-checking, or a hash of the PUF value is sent to a blockchain for identification purposes. Second, the blockchain generates and sends a challenge to the IoT device. The challenge is fed to the RC network, and the output is sent back to the blockchain for verification. Our proposed PUF employs the first method because the challenge-response method has two main disadvantages. Achieving one-way communication with the blockchain is easier for resource-constrained IoT devices, while the challenge-response method requires two-way communication. In the challenge-response method, correct challenge-response pairs must be stored somewhere (either third-party storage or the blockchain), creating an additional attack vector. On the other hand, local measurement and usage of the PUF value eliminate such attack vectors.

The proposed PUF value is measured as follows:

- The pull-up resistors are enabled, and capacitors *C*_1_ and *C*_2_ are charged to voltage values determined by the resistances of the internal pull-up resistors (*R_PU_*_1_ and *R_PU_*_2_) and external resistors (*R*_1_ and *R*_2_). Resistor *R*_1_ should have a lower resistance than *R*_2_ so that the voltage across capacitor *C*_1_ is lower than that across capacitor *C*_2_.
- A timer is started when the pull-up resistors are disabled, and the charge stored in capacitors *C*_1_ and *C*_2_ starts to deplete through resistors *R*_1_ and *R*_2_, respectively. The capacitances of capacitors *C*_1_ and *C*_2_ should be chosen so that the time constant *R*_1_*C*_1_ is larger than the time constant of *R*_2_*C*_2_. This ensures that capacitor *C*_1_ discharges slower than capacitor *C*_2_.
- The voltage values across capacitors *C*_1_ and *C*_2_ are monitored by an analog comparator, a built-in hardware feature found in most microcontrollers. When the voltage across capacitor *C*_2_ drops below that across capacitor *C*_1_, the analog comparator generates an interrupt that stops the timer.
- The timer value corresponds to the proposed PUF value.

**Figure 2:**
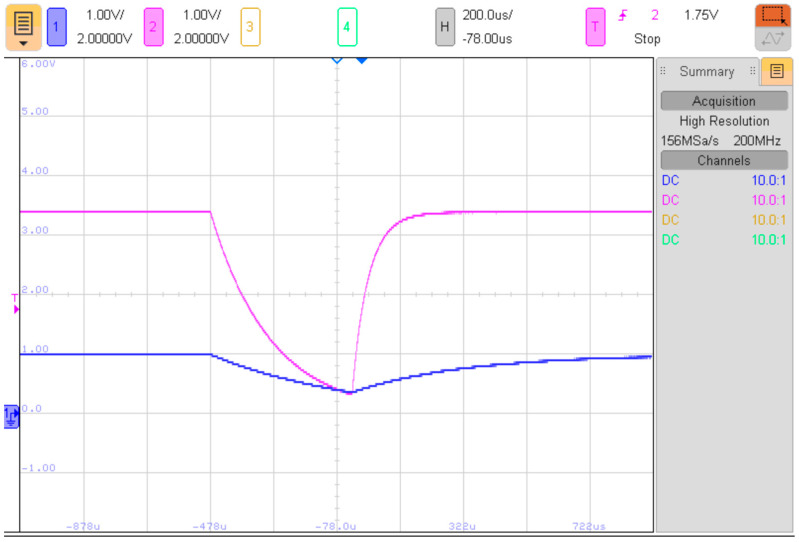
Voltage waveforms for a PUF measurement, *U_C_*_1_ (blue trace), *U_C_*_2_ (purple trace), *R*_1_ = 10 kΩ, *C*_1_ = 47 nF, *R*_2_ = 100 kΩ, *C*_2_ = 2 nF.

The voltage waveforms of a single PUF measurement are illustrated in Figure 2. The component values used in the RC networks are *R*_1_ = 10 kΩ, *C*_1_ = 47 nF, *R*_2_ = 100 kΩ, *C*_2_ = 2 nF. With a supply voltage of 5 V, the maximum voltage across capacitors *C*_1_ and *C*_2_ is 1 V and 3.4 V, respectively. The time interval that determines the PUF value is approximately 450 µs. During this interval, the timer counts several clock pulses, which becomes the proposed PUF value. After the PUF measurement, the timer is reset to zero, and the pull-up resistors are re-enabled to allow the capacitors to recharge. The capacitor recharging can be observed in Figure 2 as rising edges of the blue and purple traces.

**Figure 3:**
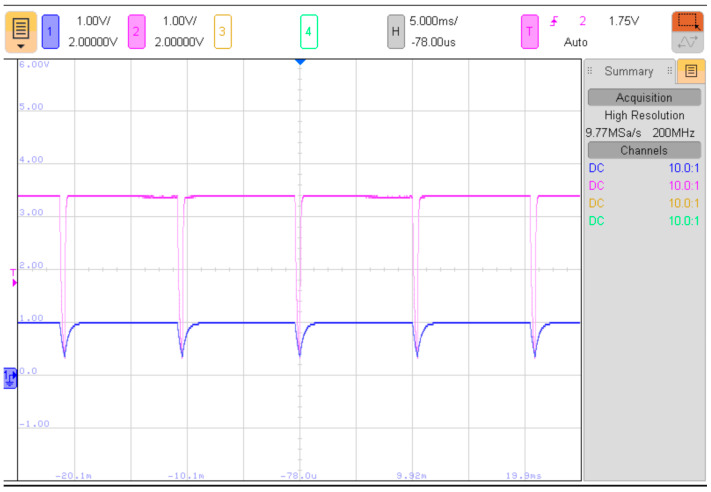
Voltage waveforms during consecutive PUF measurements, *U_C_*_1_ (blue trace), *U_C_*_2_ (purple trace), *R*_1_ = 10 kΩ, *C*_1_ = 47 nF, *R*_2_ = 100 kΩ, *C*_2_ = 2 nF.

To ensure that the capacitors are fully recharged between consecutive PUF measurements, a sufficient time interval for recharging should be provided. Figure 3 depicts the voltages across the capacitors during consecutive PUF measurements with a recharging time interval of approximately 10 ms. As mentioned earlier, the steady-state voltage values of the capacitors (while the pull-up resistors are enabled) differ due to the variations in the resistive dividers formed by *R_PU_*_1_-*R*_1_ and *R_PU_*_2_-*R*_2_.

## 3. Measurements

**Figure 4:**
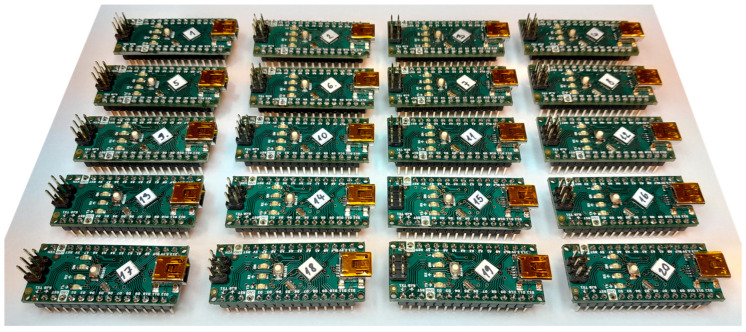
Devices under test (DUTs) are used for measurements.

**Figure 5:**
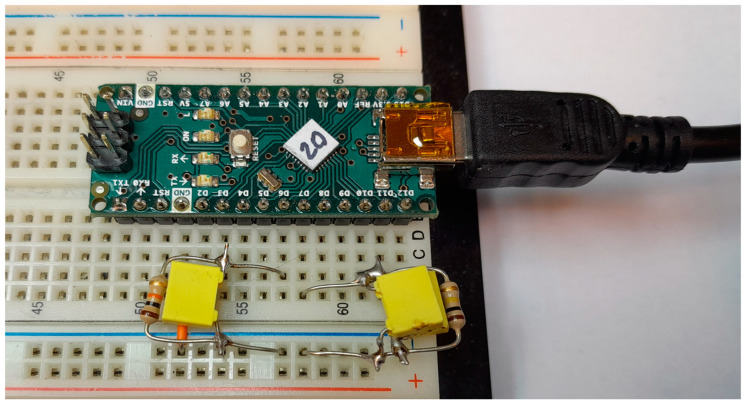
Device #20 is connected to the *RC* network on the protoboard.

Measurements were conducted on 20 identical embedded boards equipped with Atmega328P microcontrollers, as shown in Figure 4. The testing aimed to determine if the proposed PUF could successfully identify a specific device, i.e., a specific microcontroller chip. All devices were connected to the same *RC* network for testing, as illustrated in Figure 5.

**Figure 6:**
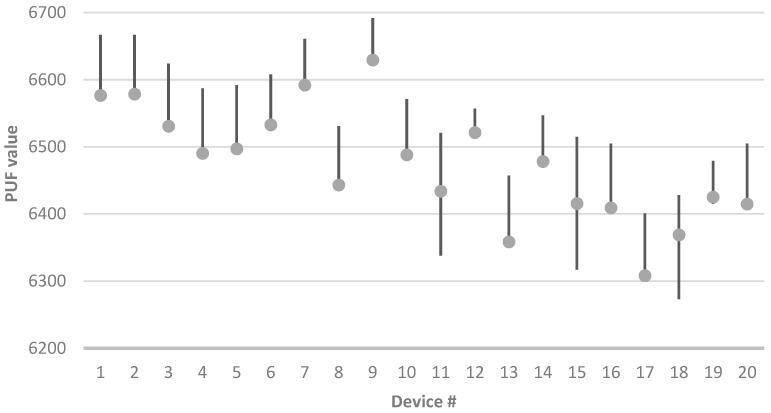
PUF value range (line) and average value (dot) for each DUT with 1000 PUF measurements.

In the first test, the PUF value was measured 1000 times for each device under test (DUT). The results are presented in Figure 6. Each device is represented by a dot on the horizontal axis, indicating the average PUF value obtained from the 1000 measurements. Additionally, a vertical line is shown to depict each device’s range of measured PUF values. It can be observed that the average value for most devices (though not all) falls towards the lower end of the measured range. Furthermore, the measured range varies among the different devices.

**Figure 7:**
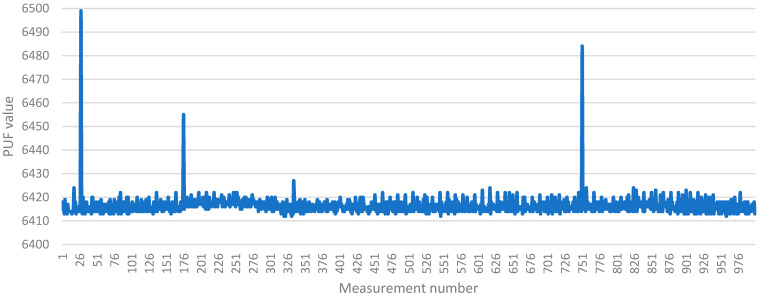
Set of 1000 PUF measurements for a single device.

To provide further insight into this behavior, Figure 7 illustrates the set of 1000 measurements obtained for a single device under test (DUT). Each point in the graph represents a single measurement of the PUF value. The variability in the measured values can be observed, indicating the inherent fluctuations in the physical properties that contribute to the PUF response.

Most measurements cluster closely around the average value (ranging from 6412 to 6424) in Figure 7, with a few peak values (6499, 6455, 6427, 6484) observed. In cases where the average values of the devices are situated in the middle of the measured range (e.g., device #11, #15, #18 in Figure 6), negative peak values are present, indicating values lower than the average.

These occasional unwanted peak values cannot be predicted, but their occurrence is infrequent, and they have a negligible effect on the overall average value of the measurements.

**Figure 8:**
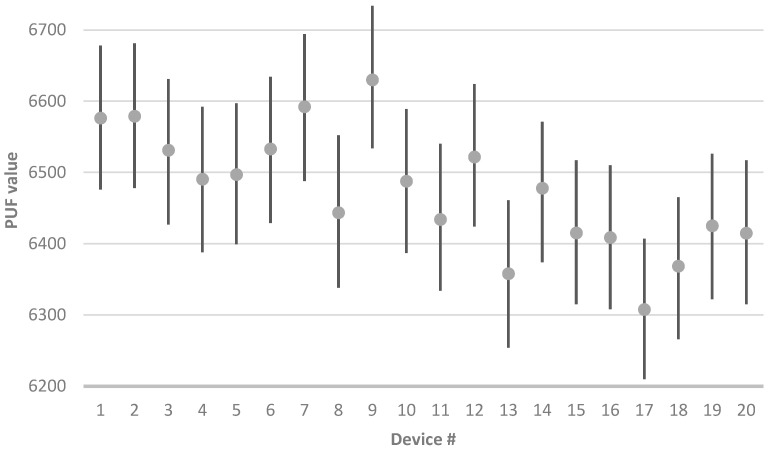
PUF value range (line) and average value (dot) for each DUT with 100,000 PUF measurements.

When conducting larger-scale measurements with 100,000 samples, positive and negative peaks are observed, with the average value centred within the measured range. In Figure 8, for 100,000 measurements, the measured range is consistent across all tested devices.

Although larger measurement sets result in improved distribution of values, conducting such measurements is time-consuming. For instance, obtaining 100,000 measurements can take approximately 15 min. In contrast, performing 1000 measurements requires around 10 s. Therefore, there is a trade-off between the level of distribution achieved and the time required to obtain the measurements.

### 3.1. Device Identification Using Proposed PUF

For the proposed PUF to effectively identify devices, each device’s PUF should be unique. In cryptographic applications, cryptographic keys with sufficiently large key spaces are commonly used. However, in the case of PUF values, which are inherently analog, the PUF value space is significantly smaller than the cryptographic essential space. In the laboratory testing of the devices, the average PUF values ranged from 6305.03 to 6633.05.

The resolution and precision of the PUF values are important parameters that define the PUF value space, i.e., the number of unique PUF values. Table 1 illustrates the impact of averaging measured PUF values on the resolution and precision of the PUF values. When a single PUF value measurement is used (batch size of 1 in Table 1), the measured values range from 6413 to 6499 (out of 100 measurements). With a single measurement, the relative error of the PUF value for a device is 1.34%. While this may seem acceptable for an analog PUF, it would perform poorly when unique device identification is required. Out of the 20 devices included in the laboratory testing, nine had their average PUF value, and 18 had their peak PUF value fall within the same range, as shown in Table 1 (6413–6499). Such resolution and precision of the PUF value are insufficient for reliable device identification.

To obtain PUF values with higher resolution and precision, batch measurements were employed, with batch sizes of 10, 100, and 1000 being evaluated. For example, with a batch size of 10, 10 single PUF measurements were taken, and the average value was calculated. The resolution and precision of the average PUF value (with a batch size of 10) are approximately an order of magnitude better than those obtained from a single PUF measurement. Larger batch sizes (100 and 1000) demonstrate even better results, with precisions of 0.0295% and 0.0159% (equivalent to an accuracy of 99.9841%). This represents an improvement of two orders of magnitude. Choosing the appropriate batch size is not straightforward and depends on the application or use case. Larger batch sizes offer higher precision and resolution but also increase the measurement time linearly (approximate measurement times for each batch size are provided in Table 1). Thus, a trade-off between precision and time should be considered to determine the most suitable batch size for a given application or use case.

**Figure 9:**
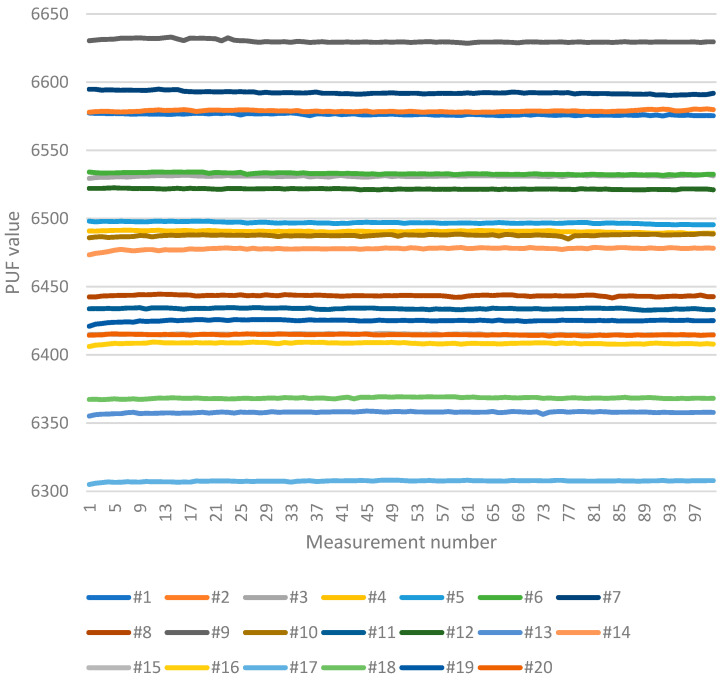
Measured PUFs for all test devices (#1–#20); 100 measurements with batch size = 1000.

This research focused on a batch size of 1000 measurements to determine the PUF values. The PUF value of each device was measured 100 times. With 20 devices included in the test, 2,000,000 single PUF measurements were conducted (20 devices × 100 PUF values × batch size of 1000). Table 2 presents the minimum and maximum values of the measured PUFs, while Figure 9 displays all the measured PUFs.

As previously mentioned, the PUF value has a certain accuracy, i.e., precision and resolution. PUF values fluctuate within a specific range of values, which becomes smaller with larger batch sizes. Table 2 provides each device’s minimum and maximum measured PUF values, corresponding to the PUFs shown in Figure 9. Among all the tested devices, only two partial overlaps in the PUF values exist. Devices #3 and #6 partially overlap for less than 30% of the PUF values. On the other hand, devices #15 and #20 overlap more significantly, accounting for up to 80% of the PUF values.

### 3.2. Tamper-Resistant Feature of the Proposed PUF

As explained in Section 3 and depicted in Figure 2, the PUF value is determined by the time it takes for the voltage across capacitor *C*_2_ (purple trace in Figure 2) to drop below the voltage across capacitor *C*_1_ (blue trace in Figure 2). Given that the PUF value is defined by a time interval, it is possible to measure the voltage waveforms across capacitors *C*_1_ and *C*_2_ to determine the PUF value of a specific device.

It is important to note that the time interval required for a single PUF measurement, as depicted in Figure 2, is fixed, and does not depend on the PUF value itself. The time interval for a single measurement is predetermined and significantly longer than the PUF value. For the PUF proposed in this paper, the time interval for a single measurement is set to approximately 10 ms, while the PUF value (in the time domain) is around 400 µs.

**Figure 10:**
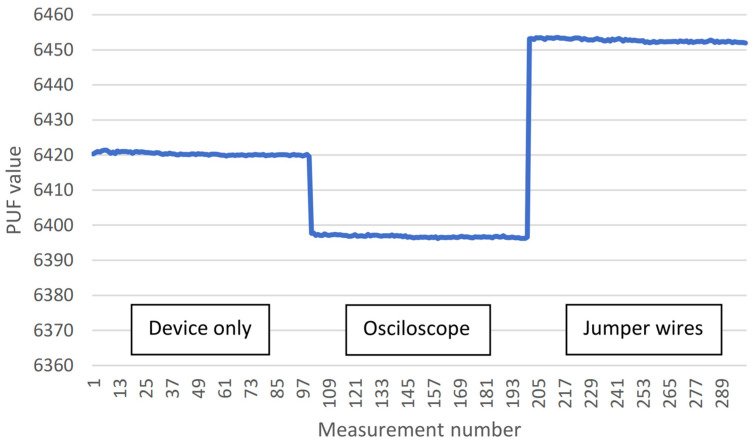
Measured PUFs for a single device (#20) in different scenarios; 100 measurements with batch size = 1000.

Table 3 presents the PUF values of a single device under three different scenarios. The first scenario represents the PUF value during normal operating conditions. The second scenario involves connecting an oscilloscope to capacitors *C*_1_ and *C*_2_ using additional jumper wires. The third scenario entails connecting only jumper wires to the device, with the other ends of the jumper wires left unconnected (“in the air”). The batch size is 1000, and the PUF measurement is repeated 100 times for each scenario. The results are also visualized in Figure 10.

These results demonstrate that the proposed PUF resists probing attempts, indicating tamper resistance. Also, the PUF is sensitive to small changes on and around the PCB, such as the addition of jumper wires, as it detects such alterations.

Comparing the PUF value for the “device only” scenario with the result for Device #20 in Table 2, a discrepancy can be observed: 6414 vs. 6420, respectively. This difference is attributed to the impact of temperature on the PUF value, a research topic planned for future work.

Cloning a device with an identical PUF value to the original device would require significant effort from an attacker. The duration of the exponential fall in Figure 2, which determines the PUF value, is determined by the *RC* network shown in Figure 1. The PUF value corresponds to the number of clock cycles from the beginning of the exponential fall to the point where the voltage across capacitor *C*_2_ drops below the voltage across capacitor *C*_1_. This time interval can be calculated using Equation (1):

(1) $$t=\frac{R_{1}C_{1}R_{2}C_{2}}{\left(R_{2}C_{2}-R_{1}C_{1}\right)}ln\left[\frac{R_{1}\left(R_{2}+R_{PU2}\right)}{R_{2}\left(R_{1}+R_{PU1}\right)}\right]$$

With a microcontroller clock frequency of 16 MHz, each microsecond is equivalent to 16 clock cycles. The PUF time interval from Figure 2 is approximately 400 µs, resulting in a PUF value of around 6400. Each PUF value has a relative resolution/precision of 0.0159% (as shown in Table 1), translating to an absolute precision of approximately one clock cycle. For an attacker to falsify a PUF value on a different device or swap the microcontroller to a different *RC* network, all external components of the *RC* network would need to be matched with an error lower than 0.02%. To put this into perspective, state-of-the-art multi-turn trimmer resistors with 25 full turns (9000°) would need to be set with precision under 2°.

The proposed PUF fingerprint offers an additional benefit over challenge-response-based PUFs. With the advancement of machine learning techniques, it has become increasingly challenging to protect challenge-response PUFs against machine learning cloning attacks [67,68]. This provides an additional reason to develop fingerprint PUFs, which are used locally and, as a result, are less susceptible to machine learning attacks.

### 3.3. Comparison with State-of-the-Art

The main contribution of the proposed PUF method is the increased accuracy and reliability achieved through the use of averaged batch measurements. This characteristic of the proposed PUF is compared with state-of-the-art methods in Table 4.

Other common metrics to evaluate PUFs are Uniqueness, Uniformity, Reliability and Bias. Uniqueness refers to the property of a PUF where each instance or individual PUF produces a unique response or output when subjected to the same input or challenge. In other words, no two devices should generate identical PUF fingerprints or identical PUF responses for the same input. Uniqueness is a crucial property that ensures the distinctiveness and non-reproducibility of each PUF, making it suitable for various security applications.

Reliability is in literature often referred to as stability or robustness. Reliability in PUFs refers to the consistency and stability of the PUF’s response over time and environmental conditions. A reliable PUF consistently produces the same response when presented with the same challenge, even with variations such as temperature changes, aging effects, or power supply fluctuations. Reliability ensures that the PUF’s behavior remains consistent and predictable over its operational lifetime.

Uniformity refers to the property of a PUF where all the individual PUF instances or units exhibit similar characteristics and behavior. In other words, PUFs should have a high degree of uniformity, ensuring that the distribution of their responses or outputs across multiple units is statistically consistent. Uniformity is essential for achieving consistent and reliable behavior across different PUF instances.

In the context of PUFs, bias refers to any systematic deviation or non-randomness in the PUF’s response. Bias can occur due to factors such as manufacturing variations, asymmetries in the PUF structure, or environmental conditions. Bias in a PUF introduces a potential vulnerability, as an adversary may exploit these deviations to predict or manipulate the PUF’s output. Therefore, minimizing bias is crucial for ensuring the security and reliability of a PUF.

The presented paper introduces a fingerprint PUF, which allows the application of uniqueness and reliability metrics. However, uniformity and bias (also known as balance or uniformity) metrics only apply to challenge-response PUF types.

Both reliability and uniqueness metrics are determined by calculating the Hamming weights of the binary responses [73]. The measured PUF values needed to be converted from decimal to binary notation to calculate these metrics. For each measured PUF value (e.g., 6419.58), we first removed the decimal point (e.g., 641,958) and converted the resulting number to binary (e.g., 10011100101110100110). This binary representation was used to calculate the PUF metrics.

Reliability is calculated using Equation (2) [73], where *T* represents the total number of samples (PUF values) measured per device, *t* is the index of a sample (1 ≤ *t* ≤ *T*), *N* is the total number of devices used, *n* is the index of a device (1 ≤ *n* ≤ *N*), *L* is the total number of bits in a device ID (device PUF fingerprint value), *l* is the index of a bit in a device ID (1 ≤ *l* ≤ *L*), and *r* is the device response.

(2) $$Reliability=1-\frac{1}{TL}\sum_{t=1}^{T}\sum_{l=1}^{L}\left(r_{n,l}⊕r_{n,t,l}\right),$$

The ideal value for the reliability metric is 100%, but the proposed PUF has a calculated reliability of 75.20%. This lower value is mainly due to the aforementioned temperature dependence. However, this temperature dependence can be utilized as a feature, as a change in the PUF value can indicate overheating of a device. This information would be beneficial for low-power devices, which typically measure ambient temperature rather than the device’s temperature, especially if they lack a dedicated temperature sensor.

Uniqueness is calculated using Equation (3) [73], and a comparison with state-of-the-art is given in Table 5.

(3) $$Uniqueness=\frac{1}{L}\frac{2}{N\left(N-1\right)}\sum_{l=1}^{L}\sum_{i=1}^{N-1}\sum_{j=i+1}^{N}\left(r_{i,l}⊕r_{j,l}\right),$$

## 4. Discussion

This paper proposes a PUF method for identity authentication and tamper resistance of IoT devices. The method leverages the high tolerance of the internal pull-up resistors in a microcontroller. Combining these resistors with an external *RC* network forms an analogue circuit, and its response is highly dependent on the selected components. To enhance the accuracy of the PUF method, the PUF value is calculated as the average value of batch measurements. By computing the average of numerous individual PUF measurements, the proposed method effectively determines the PUF value, leading to a substantial enhancement in accuracy, which is a main contribution of this work. The accuracy of the PUF value correlates with the batch size, with a trade-off of longer measurement time. This work used a batch size of 1000 measurements, taking approximately 10 s to complete on a low-cost microcontroller ATmega328P, achieving an accuracy of 99.9841% (relative error of 0.0159%).

Test results demonstrate the tamper resistance of the proposed PUF method against probing and its ability to detect even minor changes in the *RC* network, such as connected jumper wires left floating. Given the relatively long measurement time required, the proposed method is most suitable for sporadic checks, such as device self-check at startup, device identification, or payload signing during data transmission to the blockchain. The proposed PUF also exhibits temperature dependence, which can be utilized for auxiliary environmental monitoring. Still, further investigation of the temperature’s impact on the PUF value is left as future work.

The proposed PUF method offers a reliable solution for achieving identity authentication and tamper resistance in low-cost microcontrollers and IoT devices.

## Figures and Tables

**Table 1:** PUF resolution/precision.

Batch Size	Average PUF (Max. Value)	Average PUF (Min. Value)	Absolute Error	Relative Error [%]	Measurement Time
1	6499	6413	86	1.3410	≈10 ms
10	6423.1	6415.2	7.9	0.1232	≈100 ms
100	6417.75	6415.86	1.89	0.0295	≈1 s
1000	6415.45	6414.43	1.02	0.0159	≈10 s

**Table 2:** Measured PUFs for all test devices (#1–#20); 100 measurements with batch size = 1000.

Device No.	Minimal PUF Value	Maximal PUF Value
#1	6575.21	6577.5
#2	6577.83	6580.37
#3	6529.42	6532.37
#4	6488.95	6491.48
#5	6495.32	6498.12
#6	6531.63	6534.21
#7	6590.33	6594.82
#8	6441.83	6444.54
#9	6628.45	6633.05
#10	6485.05	6488.91
#11	6432.75	6434.68
#12	6520.98	6522.56
#13	6355.16	6358.86
#14	6473.38	6478.79
#15	6414.14	6415.94
#16	6406.19	6409.33
#17	6305.03	6308.24
#18	6367.21	6369.39
#19	6420.98	6425.99
#20	6413.79	6415.48

**Table 3:** Measured PUFs for a single device (#20); 100 measurements with batch size = 1000.

Scenario	Minimal PUF Value	Maximal PUF Value3
Device only	6419.58	6421.41
With oscilloscope	6396.19	6397.77
With jumper wires	6451.92	6453.54

**Table 4:** Accuracy: comparison with state-of-the-art.

PUF Design	Publication Year	PUF Accuracy
c-ROPUF [59]	2021	99.63%
XOR-mesh PUF [60]	2020	92.87%
DETTFF PUF [61]	2021	98.17%
SRAM PUF [71]	2020	97.73%
RF PUF [72]	2022	99.28%
This work	This work	99.98%

**Table 5:** Uniqueness: comparison with state-of-the-art.

PUF Design	Publication Year	Uniqueness (50% Is Ideal)
c-ROPUF [59]	2021	49.90%
XOR-mesh PUF [60]	2020	44.64%
DETTFF PUF [61]	2021	54.50%
XOR-PUF [74]	2019	47.00%
XOR FF APUF [75]	2018	43–48%
BoardPUF [76]	2015	47.21%
PCB PUF [77]	2015	47.94%
This work	This work	43.16%

## Data Availability

The data presented in this study are available on request from the corresponding author.
